# Renal thrombotic microangiopathy and nephrotic proteinuria induced by intravitreal injection of aflibercept for diabetic macular edema

**DOI:** 10.1186/s12882-022-02986-2

**Published:** 2022-10-29

**Authors:** Yawara Kikuchi, Yoshimi Odashima, Kazuhiro Yoshikawa, Tomoyasu Oda, Fumitaka Tanaka, Hiroki Oikawa, Yasushi Ishigaki, Koichi Asahi

**Affiliations:** 1 Division of Nephrology and Hypertension, Department of Internal Medicine, Iwate Medical University School of Medicine, Iwate, Japan; 2 Division of Diabetes, Metabolism and Endocrinology, Department of Internal Medicine, Iwate Medical University School of Medicine, Iwate, Japan; 3 Department of Internal Medicine, Morioka Tsunagi Onsen Hospital, Morioka, Japan

**Keywords:** Intravitreal injections, Thrombotic microangiopathy, Vascular endothelial growth factor inhibitor, Aflibercept, Diabetic macular edema, Diabetic nephropathy

## Abstract

**Background:**

Vascular endothelial growth factor inhibitors (VEGFIs) are used to treat malignant neoplasms and ocular diseases by inhibiting angiogenesis. Systemic use of VEGFIs has various side effects, including hypertension, proteinuria, and thrombotic microangiopathy, but adverse events due to intravitreal injection of VEGFIs have not been fully clarified. Although age-related macular degeneration was initially the most common target of intravitreal injection of VEGFIs, it has also been applied sporadically for diabetic macular edema in recent years. Proteinuria following intravitreal injection of VEGFIs would be reversible. In patients with diabetes mellitus (DM), however, it would be difficult to determine whether kidney damage arises from the clinical course of DM or from intravitreal injection of VEGFIs for diabetic macular edema.

**Case presentation:**

A 55-year-old woman with a 20-year history of type 2 DM began intravitreal injection of VEGFI (aflibercept, 2 mg every 4 weeks) for treatment of diabetic macular edema 2 years previously. She presented with leg edema, hypertension, and nephrotic-range proteinuria 14 months after the first injection. Histological examination of renal biopsy specimens revealed diabetic nephropathy with renal thrombotic microangiopathy probably associated with intravitreal injection of VEGFI. The patient’s nephrotic syndrome completely improved at 6 months after simply discontinuing aflibercept.

**Conclusions:**

This is a precious report of pathologically investigated renal thrombotic microangiopathy leading to nephrotic syndrome due to intravitreal injection of aflibercept for diabetic macular edema in a patient with type 2 DM. Renal function and proteinuria should be monitored in diabetic patients who receive intravitreal injection of a VEGFI. If kidney damage develops independent of the clinical course of DM during intravitreal injection of a VEGFI, renal biopsy should be performed and intravitreal VEGFI injection discontinued.

**Supplementary Information:**

The online version contains supplementary material available at 10.1186/s12882-022-02986-2.

## Background

Vascular endothelial growth factor inhibitors (VEGFIs) are used to treat malignant neoplasms and ocular diseases by inhibiting angiogenesis. Systemic use of VEGFIs is associated with various side effects, including hypertension, proteinuria, and thrombotic microangiopathy (TMA) [1–3]. In addition to promoting angiogenesis, vascular endothelial growth factor (VEGF) also maintains the morphology and function of vascular endothelial cells [1–3]. Therefore, blockade of VEGF signaling may also cause damage to glomerular vascular endothelial cells [1–3], leading to renal TMA.

VEGF levels are significantly increased in the eyeballs of patients with diabetic retinal angiopathy, which in turn promotes angiogenesis [4]. Therefore, intravitreal injection of VEGFIs is also used to inhibit VEGF signaling and prevent neoangiogenesis in those patients [4]. The intravitreal dose of VEGFI for the treatment of diabetic retinal vasculopathy is approximately 100- to 200-fold lower than the systemic dose used in the treatment of malignant neoplasms [1, 2].

Here, we report a case of nephrotic syndrome due to renal TMA that developed despite intravitreal injection of a VEGFI (aflibercept) in a female patient with diabetic macular edema (DME). Her nephrotic syndrome improved at 6 months after aflibercept discontinuation.

## Case presentation

A 55-year-old woman with a 20-year history of type 2 diabetes mellitus (DM) was treated with basal-bolus insulin and oral hypoglycemic agents. She sometimes interrupted diabetic treatment, resulting in remaining high hemoglobin A1_C_ (10%–14%) throughout her clinical course. She started intravitreal injection of aflibercept (2 mg every 4 weeks) for the treatment of DME 2 years previously. At initiation of aflibercept, her serum creatinine was 0.54 mg/dL and urinalysis showed no abnormalities. In 1- or 2-monthly monitoring of urinalysis, mild proteinuria (1 +) was found at 5 months after the first injection, but the injections were continued. Fourteen months after the first injection, she reported bilateral leg edema and hypertension (159/87 mm Hg) with a urine protein level of 4.9 g/gCr, urine red blood cells of 0–1/high-power field, and serum creatinine of 0.65 mg/dL; thus, amlodipine, telmisartan, and furosemide were added to the therapy. Nevertheless, urine protein continued to increase, to 10.0 g/gCr at 2 years after the first injection, and she was referred to our division for treatment of nephrotic-range proteinuria. A total dose of 44 mg of aflibercept was administered in 22 injections during the 2 years following the first injection, which halted the deterioration in eyesight. At the time of visiting our division, her other prescription medications included nifedipine, doxazosin mesylate, trichlormethiazide, spironolactone, rosuvastatin calcium, insulin glulisine, and insulin glargine. She had no family history of kidney disease or TMA.

The patient’s height was 155 cm, weight 64.3 kg, blood pressure 168/87 mm Hg, pulse 81/ min, O_2_ saturation 96% with room air, body temperature 37.4℃, and bilateral leg edema was noted. Computed tomography showed bilateral pleural and peritoneal effusions, but the kidneys were of normal size. Laboratory data revealed the following: white blood cell count, 4.59 × 10^3^/μL; hemoglobin, 8.2 g/dL; platelet count, 21.3 × 10^4^/μL; urine protein, 12.0 g/gCr; urine red blood cells, 10–19/high-power field; serum creatinine, 1.20 mg/dL; estimated glomerular filtration rate (using the 2021 CKD-EPI creatinine equation), 53.5 mL/min/1.73m^2^; serum albumin, 2.3 g/dL; hemoglobin A1_C_, 8.0%. No abnormalities were observed in IgG, IgA, IgM, C3, C4 levels, or autoantibodies. She had severe grade diabetic complication, such as proliferative diabetic retinopathy and diabetic neuropathy.

**Fig. 1:**
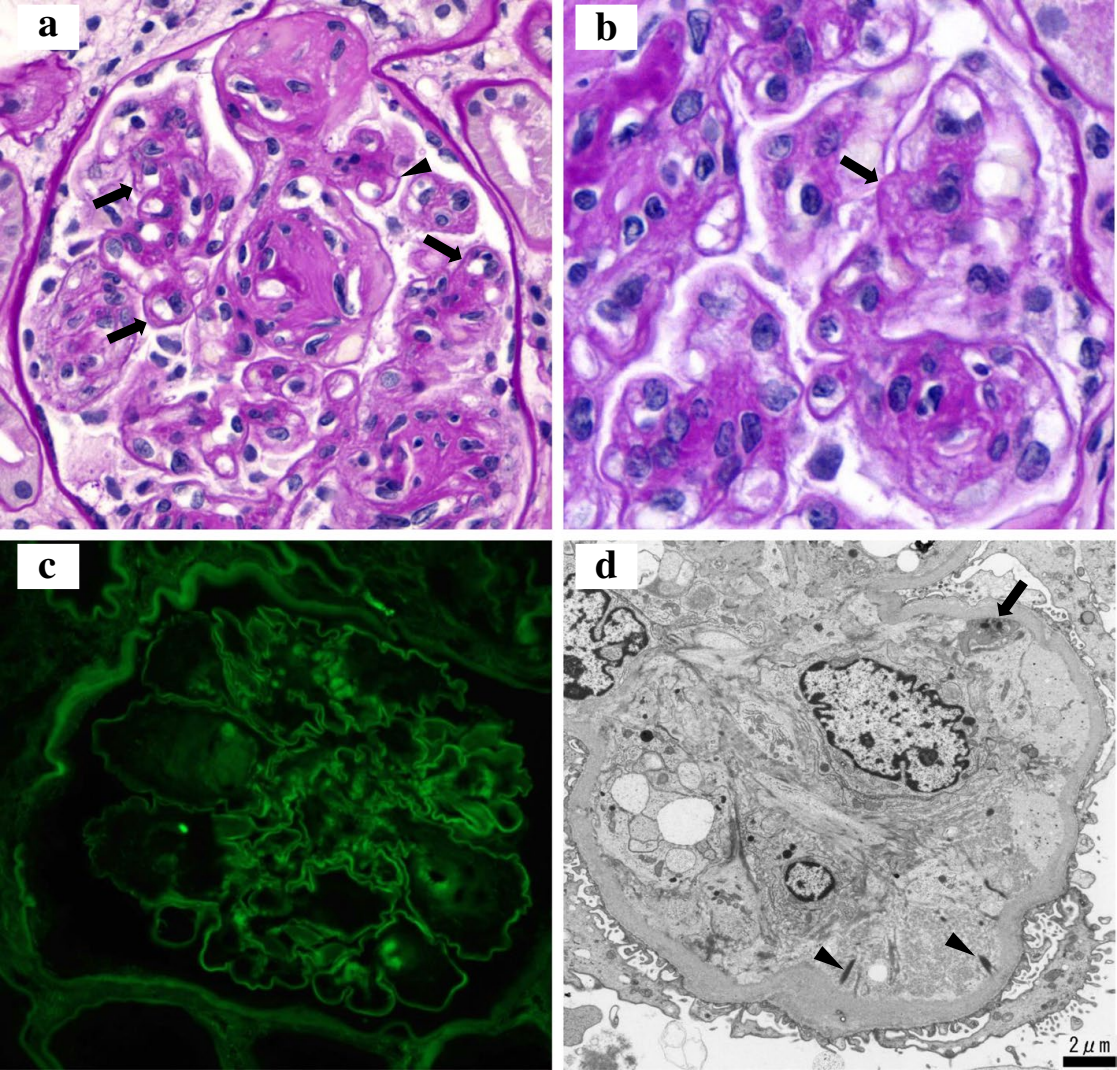
Renal biopsy findings. **a** A light microscopy showing a glomerulus with nodular lesions, duplication of the glomerular capillary wall (arrows) and expansion of the subendothelial space (arrowhead) (periodic acid-Schiff stain, original magnification × 500). **b** Mesangiolysis (arrow) in the glomerulus (periodic acid-Schiff stain, original magnification × 800). **c** Immunofluorescence staining for IgG showing linear pattern along the glomerular capillary walls (original magnification × 400). **d** Electron microscopy showing expansion of the subendothelial space with fibrin tactoids (arrowheads) and platelet (arrow) (original magnification × 4000)

Two renal biopsy specimens were obtained for histological examination. There were 32 glomeruli in the light microscopic specimens, five of which were globally sclerotic. Glomeruli showed diffuse increase of mesangial matrix with nodular lesions (Fig. 1a). Mesangiolysis (Fig. 1b, arrow), duplication of the glomerular capillary wall (Fig. 1a, arrows), and expansion of the subendothelial spaces (Fig. 1a, arrowhead) were also present in the glomeruli. There were no definite thrombi in the glomeruli. Interstitial fibrosis, interstitial inflammation, and tubular atrophy were mild. Arterial sclerosis was mild, and arteriolar hyalinosis was moderate. No narrowing of the lumen or thrombosis were observed in the interstitial arterioles. Immunofluorescence studies showed linear IgG staining along the glomerular capillary walls (Fig. 1c; IgG 1 + , intensity) and negative staining for IgA, IgM, C3, and C1q. Electron microscopy revealed diffuse thickening of the glomerular basement membrane (GBM) and increased deposition of mesangial matrix in the glomeruli. Mesangiolysis, duplication of the GBM, and expansion of the subendothelial spaces were also seen (data not shown). Some expanded subendothelial spaces contained fibrin tactoids (Fig. 1d, arrowheads) and platelets (Fig. 1d, arrow). There were no electron-dense deposits in the glomeruli and some endothelial swellings were observed in the interstitial arterioles. Collectively, these findings suggested a final diagnosis of diabetic nephropathy with renal TMA probably associated with VEGFI (aflibercept).

Intravitreal aflibercept injection was discontinued after the renal biopsy, then both urinary protein and serum albumin gradually improved. Six months after withdrawal of aflibercept, the patient no longer had nephrotic syndrome as indicated by improvement of leg edema and disappearance both of the bilateral pleural effusion and ascites. Ten months after ceasing aflibercept, her laboratory parameters (serum creatinine, 1.49 mg/dL; serum albumin, 4.0 g/dL; urine protein, 0.6 g/gCr) and blood pressure (123/54 mm Hg) had improved. Her eyesight maintained stable even after aflibercept discontinuation.

## Discussion and conclusions

In animal studies, VEGFI binding was detected in the glomeruli of monkeys after intravitreal injection of VEGFI [5]. Although experimental studies did not demonstrate a significant association between intravitreal injection of VEGFIs and renal dysfunction or proteinuria [6–9], some clinical studies suggesting an association between VEGFIs and renal dysfunction or proteinuria are beginning to appear [10, 11]. Hypertension has not been clearly demonstrated as a side effect of intravitreal injection of VEGFIs; however, there has been a recent increase in positive clinical studies reporting a relationship between them [6, 7, 12–15]. The associations of intravitreal injection of VEGFIs with increased risk of cerebrovascular events and mortality have also been controversial [16–20]. Therefore, adverse events due to intravitreal injection of VEGFIs have yet to be fully clarified.

In 2011, Pellé et al. first reported the pathology of renal biopsy (renal TMA) after intravitreal injection of VEGFI [21]. To date, 18 renal biopsies have been reported following intravitreal injection of VEGFIs (Table 1) [21–34]. In Japan, ranibizumab and aflibercept are used as intravitreal VEGFIs for age-related macular degeneration (AMD) and DME. Intravitreal injection of bevacizumab is also performed as an off-label use. AMD was initially the most common disease targeted by intravitreal injection of VEGFI, and its additional application for DME has been sporadic in recent years. We found more reports of renal biopsy after the use of intravitreal bevacizumab than those of ranibizumab and aflibercept, which may suggest that intravitreal bevacizumab has higher potency, longer half-time, and stronger systemic absorption [35, 36]. The treatment burden of patients with DME has been heavy with increasing number of diabetic patients [37], and the use of VEGFI for DME is also possibly increasing. Renal TMA was the most common renal pathology among the 18 renal biopsies, being reported in 9/18 patients (Nos. 1, 4, 9, 10, and 14–18 in Table 1) [21, 23, 28, 29, 32–34]. Of these, only three patients showed improvement after discontinuation of intravitreal injection of VEGFI for AMD or glaucoma (Nos. 1, 9 and 18 in Table 1) [21, 28, 34]. In our case, renal TMA developed due to intravitreal aflibercept injection for DME—not for AMD or glaucoma—and the condition improved after simply discontinuing intravitreal injection of aflibercept, without the use of corticosteroids or other drugs.

The glomerular changes in the present case are consistent pathologically with diabetic nephropathy (class III); however, considering the doubling of the glomerular capillary wall (basement membrane) and expansion of the subendothelial space, the morphological findings are also compatible with renal TMA [38]. In fact, the improvement of proteinuria after discontinuing intravitreal aflibercept suggests that the primary cause of proteinuria was renal TMA due to intravitreal aflibercept rather than coexisted diabetic nephropathy.

There have been reports of TMA improvement after discontinuing of intravitreal injection of VFGFI for AMD and glaucoma [21, 28, 34]. To the best of our knowledge, this is a precious report of renal TMA that developed due to intravitreal injection of aflibercept for DME in a patient with type 2 DM, which improved simply by discontinuing aflibercept, without the use of corticosteroids or other drugs.

Additionally, there have been several reports of the occurrence of clinical symptoms after the initiation of intravitreal injections of VEGFI, although histopathological examinations of renal biopsy specimens were not performed [1, 2]. The most common condition was worsening eGFR, others were proteinuria, hypertension, hypertensive cerebral hemorrhage, and relapsed minimal change disease [1, 2, 26, 30].

Kidney damage associated with DM is often irreversible [39]. However, proteinuria due to intravitreal injection of VEGFI may be reversible. In patients with a long history of DM, it would be difficult to determine whether the kidney damage is due to DM or intravitreal injection of VEGFI in the clinical course. If kidney damage develops after starting intravitreal injection of VEGFI, we recommend performing histopathological examination of renal biopsy and discontinuing VEGFI immediately.

This patient had no familial history of TMA and did not present clinical features or autoantibodies suggesting autoimmune disease. VEGFI-induced renal TMA rarely develops the classical hematologic abnormalities found in acute general TMA [40]. Our report is, however, limited by not confirming serum aflibercept level, serum VEGF level, haptoglobin level, and ADAMTS13 level. Nevertheless, we consider that it does not take away the merit of this case report, since it included a histopathological examination of renal biopsy specimen. In the future, repeat renal biopsy proving the disappearance of the TMA would further support our conclusion.

We reported a case of renal TMA that had probably been induced by intravitreal injection of aflibercept for DME. This is a precious report of pathologically investigated renal TMA due to intravitreal injection of aflibercept for DME in a patient with a history of type 2 DM, which improved simply by discontinuing intravitreal injections of aflibercept, without the use of corticosteroids or other drugs. It is therefore necessary to perform careful monitoring of the development of renal damage during intravitreal injection of aflibercept.

## Supplementary Information

**Additional file 1:** **Table 1. **Summary of literature on renal biopsy after intravitreal injection of vascular endothelial growth factor inhibitors.

## Data Availability

Not applicable.

## Abbreviations

ADPKD: Autosomal dominant polycystic kidney disease
Aflib: Aflibercept
AIN: Acute interstitial nephritis
AMD: Age-related macular degeneration
Bev: Bevacizumab
BPH: Benign prostatic hyperplasia
C1q: Complement component 1q
C3: Complement component 3
C4c: Complement component 4
CAD: Coronary artery disease
CC: Corticosteroids
cFSGS: Collapsing focal and segmental sclerosis
CKD: Chronic Kidney Disease
CKD-EPI: Chronic Kidney Diseases – Epidemiology Collaboration
COPD: Chronic obstructive pulmonary disease
CyA: Cyclophosphamide
DM: Diabetic mellitus
DME: Diabetic macular edema
DN: Diabetic nephropathy
DR: Diabetic retinopathy
ESRD: End-stage renal disease
FPI: Fresh plasma infusion
GBM: Glomerular basement membrane
HD: Hemodialysis
HNS: Hypertensive nephrosclerosis
HT: Hypertension
IgA: Immunoglobulin A
IgG: Immunoglobulin G
IgM: Immunoglobulin M
IVIG: Intravenous immunoglobulin
M: Months
MD: Macular degeneration
MGN: Membranous glomerulonephritis
MMF: Mycophenolate mofetil
NR: Not recorded
Paf: Paroxysmal atrial fibrillation
PE: Plasma exchange
PSL: Prednisone
Ran: Ranibizumab
SSc: Systemic sclerosis
T1DM: Type 1 diabetic mellitus
T2DM: Type 2 diabetic mellitus
TMA: Thrombotic microangiopathy
VEGF: Vascular endothelial growth factor
VEGFI: Vascular endothelial growth factor inhibitor
VEGFIs: Vascular endothelial growth factor inhibitors
Y: Years
