# Correction: Renal thrombotic microangiopathy and nephrotic proteinuria induced by intravitreal injection of aflibercept for diabetic macular edema

**DOI:** 10.1186/s12882-022-03006-z

**Published:** 2022-11-28

**Authors:** Yawara Kikuchi, Yoshimi Odashima, Kazuhiro Yoshikawa, Tomoyasu Oda, Fumitaka Tanaka, Hiroki Oikawa, Yasushi Ishigaki, Koichi Asahi

**Affiliations:** 1 Department of Internal Medicine, Division of Nephrology and Hypertension, Iwate Medical University School of Medicine, Iwate, Japan; 2 Department of Internal Medicine, Division of Diabetes, Metabolism and Endocrinology, Iwate Medical University School of Medicine, Iwate, Japan; 3 Department of Internal Medicine, Morioka Tsunagi Onsen Hospital, Morioka, Japan

**Correction: BMC Nephrol 23, 348 (2022)**

**https://doi.org/10.1186/s12882-022-02986-2**

Following publication of the original article [1], the authors have replaced the Supplementary Information (Additional file 1).

The original article has been corrected.

## Supplementary Information

**Additional file 1 Table 1.** Summary of literature on renal biopsy after intravitreal injection of vascular endothelial growth factor inhibitors.
